# Impact of a spatial repellent product on *Anopheles* and non-*Anopheles* mosquitoes in Sumba, Indonesia

**DOI:** 10.1186/s12936-022-04185-8

**Published:** 2022-06-03

**Authors:** Dendi H. Permana, Siti Zubaidah, Lepa Syahrani, Puji B. S. Asih, Din Syafruddin, Ismail E. Rozi, Anggi P. N. Hidayati, Sully Kosasih, Farahana K. Dewayanti, Nia Rachmawati, Rifqi Risandi, Michael J. Bangs, Claus Bøgh, Jenna R. Davidson, Allison L. Hendershot, Timothy A. Burton, John P. Grieco, Evercita C. Eugenio, Fang Liu, Nicole L. Achee, Neil F. Lobo

**Affiliations:** 1 Eijkman Institute for Molecular Biology, National Research and Innovation Agency, Diponegoro 69, Jakarta Pusat, 10430 Indonesia; 2 Department of Parasitology, Faculty of Medicine, Hasanuddin University, Makassar, Indonesia; 3 Public Health and Malaria Control, PT Freeport Indonesia, International SOS, Kuala Kencana, Papua, Indonesia; 4 Department of Entomology, Faculty of Agriculture, Kasetsart University, Bangkok, Thailand; 5 The Sumba Foundation, Public Health and Malaria Control, Bali, Indonesia; 6 Department of Biological Sciences, Eck Institute for Global Health, University of Notre Dame, Notre Dame, IN USA; 7 Sandia National Laboratories, Albuquerque, NM USA; 8 Department of Applied and Computational Mathematics and Statistics, University of Notre Dame, Notre Dame, IN USA

**Keywords:** Spatial repellent, Anthropophagic, *Anopheles*, Mosquitoes, Sumba, Indonesia

## Abstract

**Background:**

The East Nusa Tenggara province, Indonesia, contributed to 5% of malaria cases nationally in 2020, with other mosquito-borne diseases, such as dengue and filariasis also being endemic. Monitoring of spatial and temporal vector species compositions and bionomic traits is an efficient method for generating evidence towards intervention strategy optimization and meeting disease elimination goals.

**Methods:**

The impact of a spatial repellent (SR) on human biting mosquitoes was evaluated as part of a parent cluster-randomized, double-blinded, placebo-controlled trial, in Sumba, East Nusa Tenggara. A 10-month (June 2015–March 2016) baseline study was followed by a 24-month intervention period (April 2016 to April 2018)—where half the clusters were randomly assigned either a passive transfluthrin emanator or a placebo control.

**Results:**

Human-landing mosquito catches documented a reduction in landing rates related to the SR. Overall, there was a 16.4% reduction (21% indoors, and 11.3% outdoors) in human biting rates (HBR) for *Anopheles*. For *Aedes*, there was a 44.3% HBR reduction indoors and a 35.6% reduction outdoors. This reduction was 38.3% indoors and 39.1% outdoors for *Armigeres*, and 36.0% indoors and 32.3% outdoors for *Culex* species. Intervention impacts on the HBRs were not significant and are attributed to large inter-household and inter cluster variation. *Anopheles flavirostris*, *Anopheles balabacensis* and *Anopheles maculatus* individually impacted the overall malaria infections hazard rate with statistically significance. Though there was SR-based protection against malaria for all *Anopheles* species (except *Anopheles sundaicus*), only five (*Anopheles aconitus, Anopheles kochi, Anopheles tessellatus, An. maculatus* and *An. sundaicus*) demonstrated statistical significance. The SR numerically reduced *Anopheles* parity rates indoors and outdoors when compared to the placebo.

**Conclusion:**

Evidence demonstrating that *Anopheles* vectors bite both indoors and outdoors indicates that currently implemented indoor-based vector control tools may not be sufficient to eliminate malaria. The documented impact of the SR intervention on *Aedes*, *Armigeres* and *Culex* species points to its importance in combatting other vector borne diseases. Studies to determine the impact of spatial repellents on other mosquito-borne diseases is recommended.

**Supplementary Information:**

The online version contains supplementary material available at 10.1186/s12936-022-04185-8.

## Background

Mosquito borne diseases in Indonesia remain a major health problem with high morbidity and mortality. Significant mosquito-borne diseases are present in Indonesia include malaria transmitted by *Anopheles* species; dengue, Chikungunya, and Zika virus transmitted by *Aedes* species; filariasis transmitted by multiple genera of mosquito, such as *Mansonia*, *Anopheles*, *Culex*, *Aedes*, and *Armigere*s; and Japanese encephalitis (JE) transmitted by *Culex* species [1, 2].

Malaria, dengue, Zika and filariasis are endemic in many areas of Indonesia whereas Chikungunya and JE occur sporadically or as outbreaks [3, 4]. A decline in the incidence rates of dengue malaria and filariasis, but not chikungunya, have been documented since 2016. For malaria, the annual parasite incidence (API) significantly decreased from 1.96 per 1000 in 2010 to 0.93 per 1000 in 2019 [5]. The majority of malaria cases occur in the five eastern Indonesian provinces—Papua, West Papua, East Nusa Tenggara, Maluku and North Maluku. The incidence rate for dengue as of 2019 was 5153 per 100,000, while its fatality rate was 0.67% There were 10,758 filariasis cases reported in Indonesia with the highest incidence found in Papua (3615 cases), East Nusa Tenggara (1540 cases), and West Papua (1089 cases) [6]. Approximately 5042 cases of chikungunya were reported during 2019, albeit without any recorded deaths [5].

The provincial health department of East Nusa Tenggara reported in 2018 that Sumba Island had a demonstrated vector-driven health burden with four regencies contributing to malaria 69%, filaria 27% and dengue 24% to the province total. The Regional Health Ministry in Sumba has committed to eliminate these vector-borne diseases—with a focus on malaria, by 2023. Research in vector control would enable this province to meet the elimination target by providing evidence for intervention and strategy optimization [7].

Although malaria vector control, primarily through the use of long-lasting insecticidal nets (LLINs) and indoor residual spraying (IRS) [8], has led to a reduction of malaria cases worldwide. [9], the World Health Organization (WHO) reported a stall in progress in 2017 [10]. LLINs impact late night and indoor feeding vectors, whereas IRS targets those that rest indoors [11]. Mosquito behaviours that allow vectors to avoid interventions (such as early evening and outdoor resting) as well as the emergence and spread of insecticide resistance, result in gaps in protection where transmission may continue. Alternative and innovative vector control tools are required to combat diseases in these spaces and times.

Spatial repellency (SR) is used to refer to a range of insect behaviours induced by airborne, volatile chemicals that ultimately result in a reduction in human-vector contact [12, 13]. These behaviours include movement away from a spatial repellent treated space with chemical stimulus, interference with host detection (attraction-inhibition) and/or interference with feeding response (feeding-inhibition) [14–17]. The SR paradigm may complement present intervention strategies and further reduce disease incidence. Spatial repellent products may protect humans not only from malaria, but also the other vector borne diseases as well as nuisance mosquitoes. With regards to malaria, a spatial repellent product was demonstrated to have a protective efficacy against malaria infection and reduces the attack rate of the primary vector *Anopheles sundaicus* sensu lato (*s.l*.) by 32.9% in Sumba, Indonesia [18]. In addition, the protective effect of a SR intervention on malaria incidence has been reported in this parent epidemiological trial [19].

This study evaluates the impact of the spatial repellent product on both *Anopheles* and non-*Anopheles* landing rates, among other endpoints, in a field setting over the course of the parent study baseline and intervention period.

## Methods

The study represents the entomological component of the parent clinical trial registered in clinical trials.gov (Identifier: NCT02294188) and performed according to Good Laboratory Practice (GLP) and Good Clinical Practice (GCP) guidelines [19].

### Site description

The study was conducted in Southwest and West Sumba Districts, East Nusa Tenggara Province, Indonesia, as part of a larger epidemiological trial evaluating the effect of a spatial repellent product on malaria [19]. Sumba island is part of the Lesser Sunda Archipelago, located in East Nusa Tenggara Province, Indonesia, at 9° 40ʹ S, 120° 00ʹ E. The island is divided into four districts; Southwest Sumba, West Sumba, Central Sumba and East Sumba Districts. The population of Southwest and West Sumba is approximately 448,750 in 2020 [20]. Most residents are subsistence farmers. The climate is tropical, with a drier season from May to November and a wetter season from December to April. Traditional Sumba houses have a square layout without windows. Cross ventilation and, therefore, mosquito entry, is enabled by gaps in the wall, which are made from plaited palm fronds.

### Entomologic surveys

**Fig. 1:**
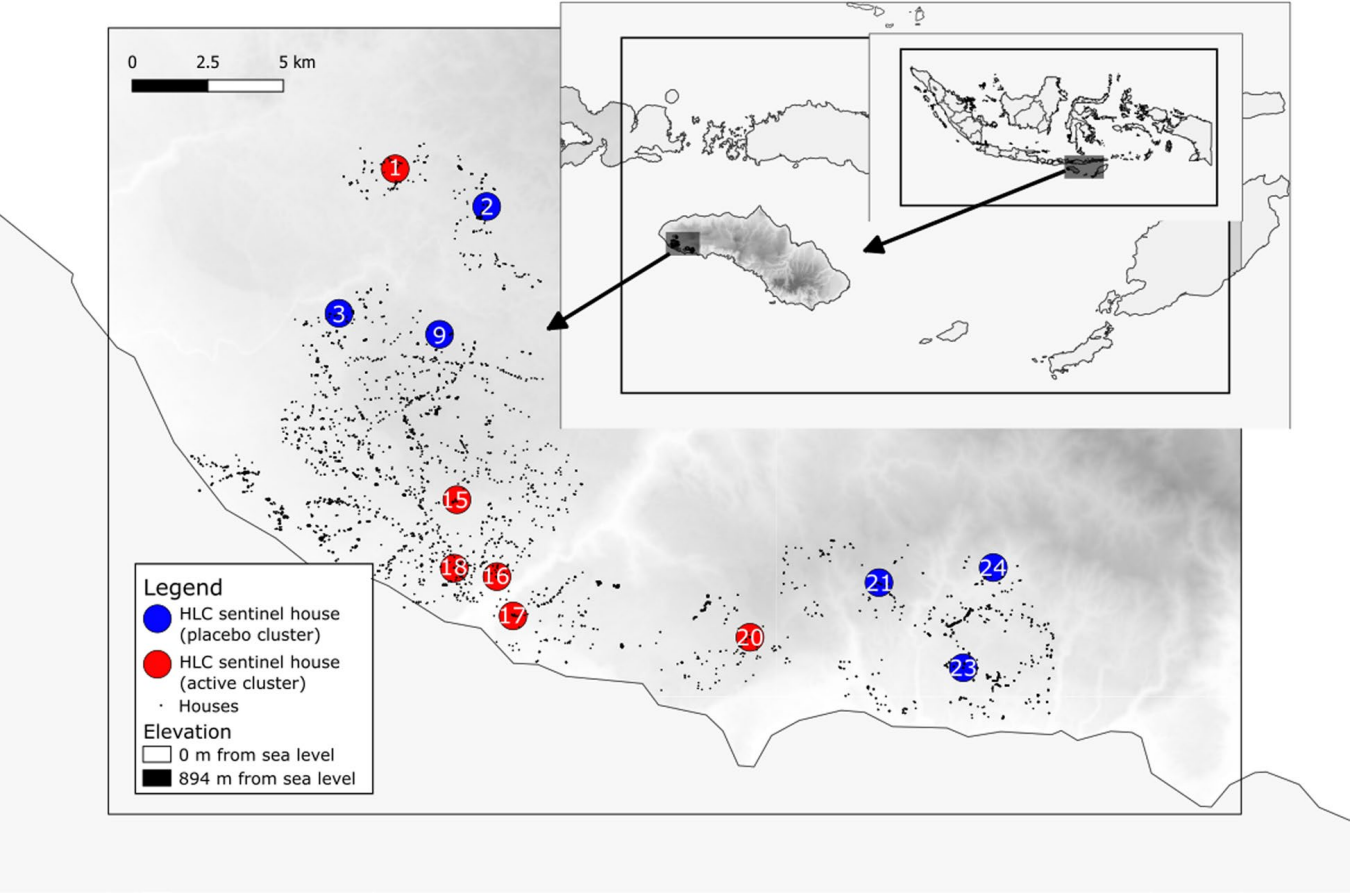
Map of the study site: sentinel house clusters are marked by circle in Sumba (larger inset), Indonesia (small inset). Spatial repellant (active) clusters 1, 15, 16, 17, 18 and 20 (in red), while placebo clusters 2, 3, 9, 21, 23 and 24 are in blue. Map from Natural Earth. https://www.naturalearthdata.com/

This parent study [19] is a cluster-randomized, double-blinded, placebo-controlled trial involving 24 clusters of households. Each cluster has approximately 100 houses (Fig. 1). Adult human biting mosquito diversity and density were measured using human-landing catches (HLC) [21]. Every 2 weeks from the start of the baseline (May 2015 to May 2016) through the end of the intervention period (April 2016 to April 2018) in a subset of 12 clusters. For the intervention trial, clusters for entomological sampling were hierarchically stratified based on human landing rate and blindly allocated to treatment arm to ensure a balanced recruitment (six clusters in each treatment group). For all collections, four neighbouring sentinel houses within each of the 12 clusters were selected for mosquito collections (n = 48). Collections were conducted at sentinel houses for one night every two weeks from paired active/placebo clusters (e.g., three pairs on Monday night and three pairs on Wednesday night). All mosquito collectors were trained, with competency being assessed before entomological sampling. Each collector provided informed consent at the beginning of the study. Teams of two collectors were assigned per house, one positioned indoors near the centre of the house and one located outside on the house veranda, approximately ~ 1 m from the exterior wall. Paired collectors rotated their positions (indoor and outdoor) every hour towards reducing individual sampling biases. HLC collectors removed all mosquitoes landing on their exposed lower legs using a mouth aspirator. Collections were conducted from 18.00 to 06.00 h for 50 min every hour. Samples were placed into individual holding containers labelled by collection hour interval, the sentinel house hold code, and collection location (indoor or outside). Captured mosquitoes were immediately killed by organic compound vapor in the field and initially identified to species (or species complex) using morphological characteristics.

### Species identification

All mosquitoes collected during HLCs were transferred to a field laboratory for morphological identification using illustrated keys for adults and larval stages [22]. A subset of morphologically identified samples were molecularly identified by sequencing the ITS2 and/or cytochrome oxidase I (COI) gene [23–26]. Samples processed were randomly chosen to represent all morphologically identified specimens, geographies and time periods of collection. PCR amplicons were sequenced at the Eijkman Institute for Molecular Biology, Indonesia, and the University of Notre Dame, USA.

Primary analysis consisted of *Anopheles* species-specific landing rates between the baseline and intervention periods as well as between intervention and placebo arms of the spatial repellent intervention study [19]. Analysis of the effect of the spatial repellent intervention on non-*Anopheles* was based on the genus level.

### Effects of SR on anopheles HBR, parity/nulliparity rates, and sporozoite positivity rate

The frequency and percentage for each of the *Anopheles* species detected based on morphological identification were summarized by treatment (SR versus placebo) and collection location (indoor versus outdoor) for the baseline and the intervention periods. The 10 *Anopheles* species that do not have the data parity issue (count in each treatment collected in each location at each time point > 5) in the frequency analysis were analysed to examine whether SR reduces the density of a particular species compared to placebo. First, the total number of each of the 10 species collected per day per household (HBR) were calculated for each collected time point (every 2 weeks) by collection location and treatment for the baseline and intervention periods. This aggregated daily HBR was then analysed via a mixed-effect negative binomial (NB) regression model. The covariates in the model include the fixed effects of treatment arm, time, collection location, cluster population size, log-scaled baseline HBR, baseline malaria incidence rate, a random effect for household within cluster to account for the temporal correlation, and a random effect for cluster to account for the correlation among households within the same cluster. The % changes in the HBR by SR compared to placebo were estimated. To control the overall Type-I error when searching for a statistically significant reduction across the species and collection location, the Benjamini-Hochberg (BH) multiplicity adjustment procedure (MCP) was applied to obtain the adjusted p-value defined as $\mathrm{min}\left\{m{p}_{\left(j\right)}/j, 1\right\}$ for each comparison, where $m$ is total number of tests, ${p}_{\left(j\right)}$ is the $j$-th smallest raw p-value among from the *m* total tests.

The frequency and percentage of parous and nulliparous mosquitoes are summarized by treatment arm and collection location for the baseline and intervention periods. Summary statistics (mean, SD, min, and max) were obtained by treatment and collection location for the parity and nulliparity rates among the daily mosquitoes collected by one person. The parity and nulliparity rate was also analysed via a similar NB regression model with the total number of mosquitoes by treatment and collection location as an offset. The % difference between SR and placebo on parity and nulliparity rates by collection location was estimated.

The frequencies of sporozoite positivity (*Plasmodium falciparum, Plasmodium vivax*, negative, or unclear) and the sporozoite positivity rate (*P. falciparum* + *P. vivax*) / (*P. falciparum* + *P. vivax* + unclear + uninfected) are summarized by treatment for the baseline and intervention periods. The time profile of the daily sporozoite positivity rate among the mosquito over the study period is plotted. Model-based analysis was not conducted for the sporozoite positivity rate due to data sparsity.

### Effects of mosquito HBR and parity/nulliparity rates on malaria incidence rates and PE of SR compared to placebo

To examine whether the frequency of any of the 10 species is associated with the first-time malaria incidence rate, a complementary log–log (cloglog) regression model on the interval-censored time-to-infection data was applied. The covariates in the model include visit, subject age, subject gender, household-level characteristics (number of doors, open eaves Y or N, wall type), and baseline incidence rate, cluster population size, treatment, collection location, the treatment and collection location interaction, mosquito frequency, and a cluster-level random effect to account for the correlation among households within the same cluster. The regression coefficient for mosquito frequency represents the log fold change in the first-time malaria rate with one unit increase in mosquito frequency. The model was repeated by the 10 species and by collection location. The BH MCP was applied to control the overall Type-I error rate. Similar cloglog regression models were applied to examine whether the parity (nulliparity) rate is associated with the first-time malaria incidence rate, replacing the mosquito frequency with parity (nulliparity) rate as a covariate in the models. The regression coefficient for parity (nulliparity) rate represents the log fold change in first-time malaria incidence rate with one unit increase in the parity (nulliparity) rate. The same set of analysis above was repeated for the overall malaria infections.

To examine whether the frequency of any of the 10 species is associated with the PE of SR against the first-time malaria infection, a similar cloglog model as the above was used, but replacing the mosquito frequency with the interaction term between mosquito frequency and treatment. The regression coefficient for the interaction term represents the log-fold change in PE with one unit increase in mosquito frequency. The BH MCP was applied to control the overall Type-I error rate The model was repeated by species and by collection location. Similar cloglog regression models were applied to examine whether the parity (nulliparity) rate is associated with the PE of SR vs placebo, replacing the interaction term between mosquito frequency and treatment with the interaction term between parity (nulliparity) rate and treatment as a covariate in the models. The same set of analysis above was repeated for the overall malaria infections.

### Non-*Anopheles* mosquitoes

In addition to the analysis of the *Anopheles* species data, non-*Anopheles* mosquito data (*Aedes* spp., *Armigeres*, and *Culex* spp.) were analysed based on how their density changed during the study and by treatment arm. The daily number of total collected mosquitoes per household by collection location (indoor versus outdoor) were calculated and then averaged across the collected time points during the baseline period and during the intervention period. The mean and standard deviation of this aggregated HBR per day per household over the 6 clusters in each of two treatment arms (SR vs. placebo) during the baseline were calculated. The aggregated HBR was also analysed via a similar NB regression model as used for analysing the *Anopheles* HBR. The % reductions in HBR by SR compared to placebo were estimated. The analysis was repeated by the non-*Anopheles* genus **i**n this study.

## Results

The parent study [19] enabled temporal and routine indoor and outdoor HLC sampling, with a total of 73,507 mosquito specimens collected across 10 months of baseline and 25 months of intervention (April 2015 to April 2018) in both intervention and placebo arms (Fig. 1). These included *Culex* (40.29%; n = 29,612), *Anopheles* (40.30%; n = 29,636); *Aedes* (12.86%, n = 9451), and *Armigeres* (6.27%; n = 4608). Unidentified female mosquitoes and *Mansonia* species were grouped together as ‘others**’** (0.27%; n = 200).

### *Anopheles* species identification

Overall, 13 species of *Anopheles* identified morphologically (with molecular confirmation [19], included *Anophele aconitus, Anopheles annularis, Anopheles balabacensis, Anopheles barbirostris s.l. (An. barbirostris *sensu stricto and *Anopheles vanderwulpi), Anopheles flavirostris, Anopheles indefinitus, Anopheles kochi, Anopheles maculatus, Anopheles subpictus, An. sundaicus s.l. (Anopheles epiroticus), Anopheles tessellatus* and *Anopheles vagus*. The most common was *An. aconitus* (20.07%), followed by *An. vagus* (15.2%) and *An. flavirostris* (14.74%) (Table 1). Analysis was based on morphological identification. The frequency (percentage) of morphologically identified *Anopheles* species are presented in see Additional file 1.

**Table 1:** The effect of the SR on the human biting rate (HBR) by Anopheles species presented only for species with non-sparse data

*Anopheles* Species	Location	SR median (min, max)	Placebo median (min, max)	% Change (95% CI)	Raw-p value	BH* adjusted p-value
*aconitus*	Indoor	0 (0.43)	0 (0.6)	282.1 (59.2, 816.7)	0.003	0.060
	Outdoor	0 (0.60)	0 (0.4)	140.0 (− 4.3, 502.0)	0.062	0.413
*annularis*	Indoor	0 (0.8)	0 (0.13)	4.8 (− 64.1, 203.2)	0.939	0.988
	Outdoor	0 (0.12)	0 (0.16)	52.8 (− 47.7, 346.3)	0.438	0.674
*barbirostris*	Indoor	0 (0.3)	0(0.10)	− 16.9 (− 75.0, 175.9)	0.762	0.953
	Outdoor	0 (0.3)	0 (0.7)	− 31.8 (− 78.7, 118.3)	0.519	0.741
*flavirostris*	Indoor	0 (0.20)	0 (0.10)	36.7 (− 31.3, 171.8)	0.373	0.622
	Outdoor	0 (0.28)	0 (0.10)	46.4 (− 26.0, 189.4)	0.273	0.840
*kochi*	Indoor	0 (0.6)	0 (0.26)	− 14.0 (− 76.4, 213.5)	0.820	0.496
	Outdoor	0 (0.4)	0 (0.40)	− 26.5 (− 79.0, 156.8)	0.630	0.965
*leucophyrus*	Indoor	0 (0.1)	0 (0.1)	− 93.2 (− 99.90, 352.2)	0.210	0.420
	Outdoor	0 (0.1)	0 (0.3)	− 97.9 (− 100.0, 89.1)	0.093	0.310
*maculatus*	Indoor	0 (0.12)	0 (0.4)	112.7 (17.1, 286.4)	0.013	0.130
	Outdoor	0 (0.14)	0 (0.3)	68.7 (− 5.7, 201.8)	0.078	0.312
*sundaicus*	Indoor	0 (0.15)	0 (0.8)	− 83.8 (− 97.8, 16.9)	0.071	0.355
	Outdoor	0 (0.18)	0 (0.5)	− 77.7 (− 97.0, 64.8)	0.141	0.353
*tessellatus*	Indoor	0 (0.9)	0 (0.47)	3.9 (− 89.7, 949.2)	0.974	0.974
	Outdoor	0 (0.6)	0 (0.42)	9.7 (− 89.3, 1021.3)	0.937	1.000
*vagus*	Indoor	0 (0.16)	0 (0.47)	100.5 (− 26.2, 444.8)	0.173	0.384
	Outdoor	0 (0.10)	0 (0.37)	113.9 (− 21.3, 481.4)	0.136	0.389

*Benjamini–Hochberg multiplicity adjustment procedure

### Impact on *Anopheles* HBR

Overall HBRs over the baseline period (11.60 bpn indoors and 12.07 bpn outdoors) were higher than those during the intervention period in both intervention (6.47 indoors and 7.29 outdoors) and placebo (8.18 indoors and 8.05 outdoors) arms [19]. Towards evaluating the biting rates in the intervention and placebo arms, both indoors and outdoors, two overall comparisons were made. When looking at indoor biting rates between the intervention and placebo arms during the intervention period, there were 21% reduction in the intervention arm. When looking at outdoor biting rates, there was a 10% decrease in biting in the intervention arm.

When comparing indoor versus outdoor biting rates in the intervention clusters during the intervention period, there were 0.89 bites indoors for every 1 bite outdoors (0.89:1; intervention indoors: intervention outdoors), versus almost equal indoor and outdoor biting in the placebo clusters—1.01 bites indoors for every 1 bite outdoors (1.01:1; placebo indoors: intervention outdoors)—demonstrating a drop in biting in the SR arm indoors and a slight increase in the SR arm outdoors—compared to the overall ratio without interventions at baseline (0.88:1).

The impact of the SR intervention (relative to the placebo) on the human biting rate (HBR, bites per person per night (bpn)) was determined for the 10 *Anopheles* species with non-sparse data (Additional file 1). These included *An. aconitus, An. annularis, An. barbirostris s.l., An. flavirostris, An. kochi, An. leucosphyrus* Group*, An. maculatus, An. sundaicus s.l., An. tesselatus,* and *An. vagus*. Each species demonstrated variable impacts of SR on HBR ranging from a reduction to an increase in landing indoors and outdoors.

Based on the BH adjusted p-values, only the indoor count of *An. aconitus* was statistically different at a false discovery rate of 10% between SR and placebo arms with a higher count in the SR arm (Table 1).

### Effect of *Anopheles* species on overall malaria incidence

Towards evaluating if a specific species impacted the malaria hazard rate in a statistically significant manner, the BH MCP was used to obtain adjusted p-values for the 10 species with non-sparse data**.** Based on these adjusted p-values (Additional file 2) three species (*An. flavirostris* indoor, outdoor and sum, *An. leucosphyrus s.l.* outdoor, and *An. maculatus* sum) individually impacted the overall malaria infections hazard rate at statistically significance with a false discovery rate of 10%.

### Relationship between *Anopheles* species and overall PE

Though all species (with the exception of *An. sundaicus s.l.*) demonstrated an SR-based increase in PE (based on % difference between arms, adjusted p-values, and a false discovery rate of 10%), only five species demonstrated statistical significance (*An. aconitus, An. kochi, An. maculatus, An. tessellatus* and *An. sundaicus s.l.*) (Table 2).

**Table 2:** The effect of per-species HBR on PE against overall malaria infections

Anopheles species	Collection location	PE difference* (%)	95% CI	Raw 2-sided p-value	BH# adjusted p-value
*aconitus*	Indoor	− 4.19	− 9.10, 0.73	0.095	0.136
	Outdoor	− 5.90	− 10.85, − 0.97	0.019	0.057
	Indoor + outdoor	− 6.75	− 11.52, − 1.95	0.006	0.026
*annularis*	Indoor	− 6.00	− 11.46, 0.48	0.033	0.090
	Outdoor	− 0.92	− 6.50, 4.65	0.744	0.797
	Indoor + outdoor	− 4.43	− 9.37, 0.52	0.079	0.125
*barbirostris*	Indoor	− 5.7	− 11.63, 0.26	0.061	0.102
	Outdoor	− 6.15	− 12.55, 0.22	0.058	0.102
	Indoor + outdoor	− 15.18	− 29.50, − 0.88	0.038	0.088
*flavirostris*	Indoor	− 1.95	− 6.80, 2.94	0.438	0.505
	Outdoor	− 2.78	− 7.73, 2.24	0.278	0.348
	Indoor + outdoor	− 3.43	− 8.57, 1.70	0.188	0.245
*kochi*	Indoor	− 8.88	− 15.24, − 2.50	0.006	0.023
	Outdoor	− 9.04	− 16.1, − 1.95	0.012	0.036
	Indoor + outdoor	− 6.20	− 12.15, − 0.26	0.04	0.086
*leucosphyrus*	Indoor	− 14.57	− 31.16, 2.08	0.086	0.129
	Outdoor	− 6.15	− 24.87, 12.52	0.519	0.577
	Indoor + outdoor	− 9.91	− 22.55, 2.69	0.124	0.169
*maculatus*	Indoor	− 7.78	− 12.96, − 2.55	0.004	0.02
	Outdoor	− 10.51	− 15.37, − 5.60	< 0.0001	< 0.003
	Indoor + outdoor	− 9.17	− 14.03, − 4.77	< 0.0001	< 0.003
*sundaicus*	Indoor	6.89	0.13, 13.61	0.046	0.086
	Outdoor	7.19	0.43, 13.96	0.037	0.093
	Indoor + outdoor	5.84	0.17, 11.53	0.044	0.088
*tessellatus*	Indoor	− 8.2	− 13.49, − 2.92	0.002	0.02
	Outdoor	− 4.19	− 6.91, − 1.55	0.002	0.015
	Indoor + outdoor	− 7.88	− 12.73, − 3.01	0.002	0.012
*vagus*	Indoor	0.39	− 4.48, 5.27	0.876	0.906
	Outdoor	− 2.14	− 7.42, 3.10	0.421	0.505
	Indoor + outdoor	0.17	− 4.62, 4.96	0.944	0.944

*Benjamini–Hochberg multiplicity adjustment procedure

^#^The PE differences are only approximate (based on first-order Taylor Expansion)

The interpretation is as follow: if a HBR increases by e^1^–1 = 1.72-folds, then the PE of SR again overall malaria infection changes by PE difference %

### Relationship between SR and *Anopheles* parity

A total of 16,675 females were dissected for parity (baseline n = 6698, intervention n = 9977) with 15,418 successful dissections (baseline n = 5920, 88.4%; intervention n = 9498, 95.12%). Analysis included the mosquitoes with unknown parity status. Mean parity rates were balanced between the SR and placebo arms at baseline with a mean indoor parity rate of 0.36 per person-day (± 0.42 SD, 0, 1 (min., max.)), a mean outdoor parity rate of 0.39 per person-day (± 0.42 SD, 0, 1 (min., max.)), a mean indoor nulliparous rate of 0.17 per person-day (± 0.30 SD, 0, 1 (min., max.)) and a mean outdoor nulliparous rate of 0.15 per person-day [± 0.28 SD, 0, 1 (min., max.)].

The SR treatment numerically reduces the parity rate for all species when compared to the placebo for both indoors and outdoors—especially the latter, and numerically increases the nulliparous rate as well for both locations. However, due to the large amount of variation, none of these changes are statistically significant (Table 3). The hazard of malaria infection increased when the parity increased, and decreased when nulliparity increased (Table 4). The highest difference in parity between active and placebo clusters was seen for was seen for *An. vagus* (50.9% parity in intervention versus 76.7% in placebo clusters; n = 1365), and *An. barbirostis* (68.5% parity in intervention versus 82.9% in placebo clusters; n = 602). Other *Anopheles* species with sampled numbers more than n = 100 varied from 1% (*An. sundaicus*) to 6.8% (*An. aconitus*) decrease in parity in the intervention arm relative to the placebo arm (Table 5).

**Table 3:** Effect of the SR on parity, nulliparity and unknown parity status during the intervention period

	Location	SR	Placebo	% Change (95% CI)
Parity rate	Indoor	0.41 ± 0.44	0.41 ± 0.45	− 10.2 (− 62.1, 113.2)
	Outdoor	0.40 ± 0.44	0.43 ± 0.45	− 25.9 (− 68.8, 75.6)
Nulliparity rate	Indoor	0.16 ± 0.29	0.12 ± 0.26	58.3 (− 37.0, 298.0)
	Outdoor	0.17 ± 0.30	0.11 ± 0.25	54.9 (− 37.6, 284.3)

**Table 4:** The relationship between parity rates and malaria infection

	Collection location	Hazard ratio	95% CI
*First-time infection*			
Parity rate	lndoor	1.006	(0.999, 1.013)
	Outdoor	1.004	(0.997, 1.010)
	lndoor + outdoor	1.006	(0.999, 1.013)
Nulliparity rate	Indoor	0.989	(0.976, 1.001)
	Outdoor	0.995	(0.984. 1.007)
	Indoor + outdoor	0.985	(0.973, 0.998)
*Overall infection*			
Parity rate	Indoor	1.003	(0.999, 1.007)
	Outdoor	1.001	(0.998, 1.005)
	lndoor + outdoor	1.005	(1.001, 1.009)
Nulliparity rate	Indoor	0.994	(0.988, 1.001)
	Outdoor	0.995	(0.989, 1.001)
	Indoor + outdoor	0.990	(0.984, 0.996)

Interpretation on hazard ratio: the hazard of malaria infection changes by (1- HR) × 100% with l% unit changes in the rate

**Table 5:** Species specific parity rates during the SR intervention implementation

Species	Total number/ Parity(%)	
	Placebo	Intervention
*Anopheles aconitus*	216/71.41	2071/78.24
*Anopheles annularis*	562/54.46	112/60.32
*Anopheles barbirostris*	513/68.54	89/82.85
*Anopheles flavirostris*	681/78.25	1007/87.22
*Anopheles indefinitus*	14/50	4/85.71
*Anopheles kochi*	647/85.59	118/91.04
*Anopheles maculatus*	133/66.79	280/67.67
*Anopheles subpictus s.l*	81/85.71	7/82.72
*Anopheles sundaicus*	95/73.68	171/74.74
*Anopheles tessellatus*	785/83.86	223/86.62
*Anopheles vagus*	921/50.9	444/76.76

### Relationship between SR and *Anopheles* sporozoite rate

The frequency of sporozoite positivity (Table 6) was calculated for both intervention and baseline periods and for both SR and placebo arms. Numerically, the sporozoite positivity rate at baseline was 0.44% (12 *P. falciparum,* 9 *P. vivax*) and 0.34% (12 *P. falciparum*, 9 *P. vivax*) in the SR and placebo arms. During the intervention period this rate was 0.14% (3 *P. falciparum,* 8 *P. vivax*) and 0.07% (6 *P. falciparum*, 1 *P. vivax*, 1 *Plasmodium inui*) in the SR and placebo arms. The sporozoite rate was less than 0.5% for both arms with no statistically significant difference between the SR arm when compared to the placebo.

**Table 6:** Frequency of sporozoite positivity status

Treatment allocation	Pf	Pv	Unclear	Uninfected	Sporozoite positivity Rate = (Pf + Pv)/(Pf + Pv + unclear + uninfected)
Baseline					
SR	12	9	0	4706	0.44%
Placebo	12	9	0	6244	0.34%
Post-intervention					
SR	3	8	0	8130	0.14%
Placebo	6	1	l	9615	0.07%

### Non-*Anopheles* species identification

Non-*Anopheles* mosquitoes sampled over the course of the study included *Culex* (40.29%; n = 29612), *Aedes* (12.86%, n = 9451), and *Armigeres* (6.27%; n = 4608). Unidentified female mosquitoes and *Mansonia* species were grouped together as ‘others**’** (0.27%; n = 200) and were not included in this analysis. A sample of morphologically identified specimens were also identified molecularly [25]. There were nine known (*Culex gelidus, Culex quinquefasciatus, Culex vishnui, Culex tritaeniorhynchus, Culex pseudovishnui, Culex bitaeniorhynchus, Culex orientalis, Culex nigropunctatus*, and *Culex. fuscochepala*) and two unidentified *Culex* species. The seven *Aedes* species identified molecularly included *Aedes albopictus* and *Aedes vexans*, while five remained unidentified. Of the three *Armigeres* species documented, *Armigeres malayi* and *Armigeres subalbatus* were identified to species, while the third was similar to *Ar. subalbatus* (*Ar. cf. subalbatus*).

### Effect of SR on non-*Anopheles* HBR

**Fig. 2:**
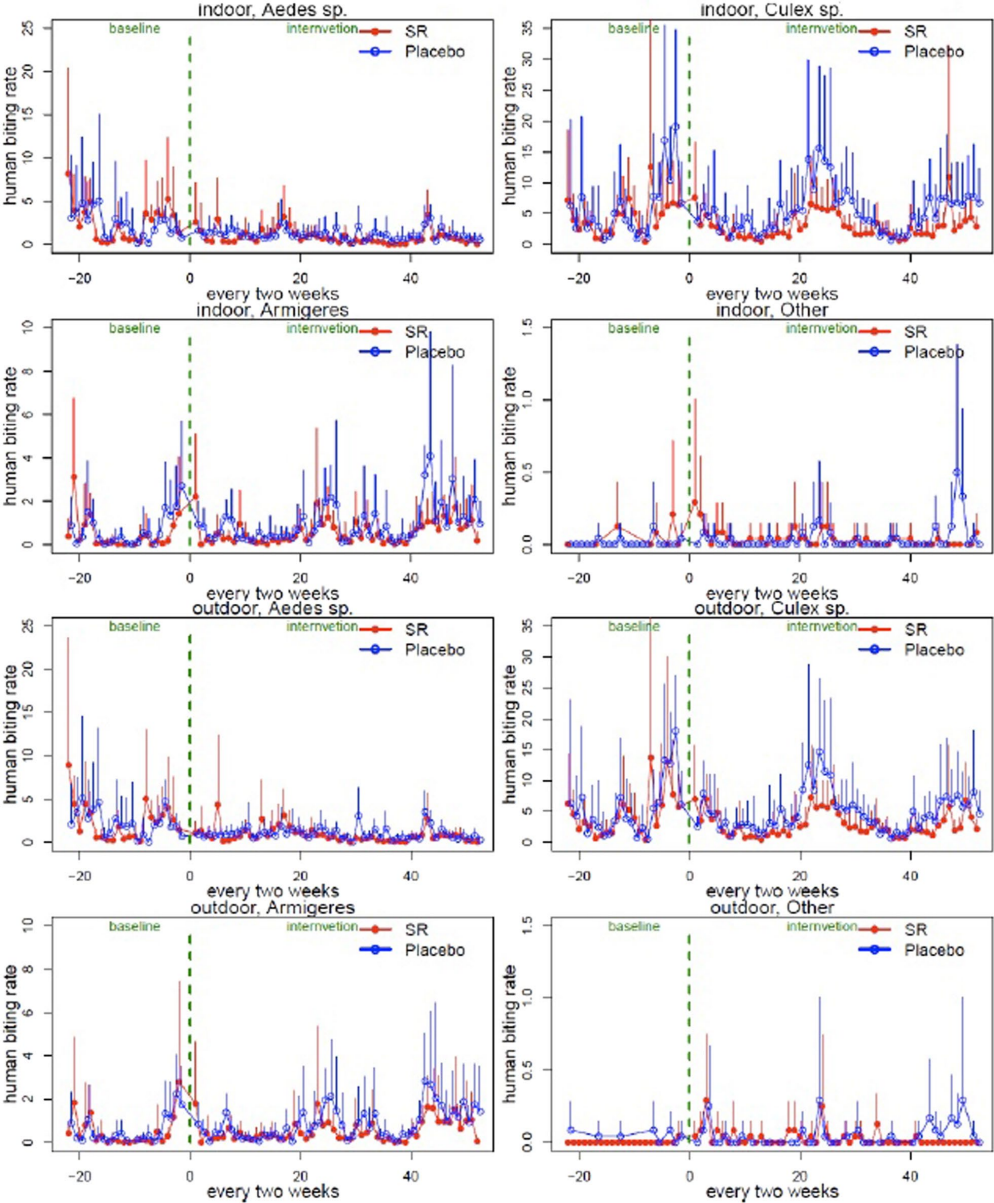
Biweekly mean (+ SD) HBR (bpn) of Anopheles mosquitoes by species, treatment and collection location

The impact of the SR intervention on non-Anopheles was restricted to biting rates for each genus. Mean HBRs were determined for *Aedes, Armigeres* and *Culex* genera for the intervention period for both SR and placebo arms over the course of the baseline and intervention period (Fig. 2).

Over the ten months of baseline, the mean per-household per-day HBR for *Aedes* was 2.36 bpn indoors and 2.28 bpn outdoors, while it was 0.56 bpn indoors and 0.49 bpn outdoors for *Armigeres*, and 5.01 bpn indoors and 4.89 bpn outdoors for *Armigeres*. The summary of the baseline non-anopheline HBR indoor and outdoor by treatment is provided in Additional file 3.

The results on the effects of SR on non-anopheline mosquitoes are presented are Table 7. There were decreases in the HBR in the SR arm when compared to the placebo across all three genera both indoors and outdoors. For *Aedes*, there was a 44.3% reduction in HBR indoors and a 35.6% reduction outdoors. This reduction was 38.3% indoors and 39.1% outdoors for *Armigeres*, and 36.0% indoors and 32.3% outdoors for *Culex* species. Due to the large variability, most of the reductions were not statistically significant.

**Table 7:** The effect of the SR on the HBR (bpn) of non-*Anopheles* mosquitoes

Species	Location	(Mean ± SD)		% Reduction (SR vs. placebo) (95% Cl)	p-Value
		SR	Placebo		
All	Indoor	4.33 ± 7.45	7.27 ± 9.26	43.1 (− 2.2, 68.3)	0.0589
	Outdoor	4.29 ± 6.25	6.59 ± 8.35	38.7 (− 10.1, 65.8)	0.1015
*Aedes* sp.	Indoor	0.86 ± 2.17	1.09 ± 1.91	44.3 (1.5, 67.0)	0.0443
	Outdoor	0.86 ± 2.21	0.99 ± 1.87	35.6 (− 14.6, 63.8)	0.1348
*Armigeres* sp.	Indoor	0.55 ± 1.48	0.90 ± 2.41	38.3 (− 37.2, 72.2)	0.2365
	Outdoor	0.13 ± 1.39	0.85 ± 2.26	39.1 (− 34.0, 72.3)	0.2178
*Culex* sp.	Indoor	2.88 ± 6.30	1.23 ± 8.34	36.0 (− 58.2, 74.1)	0.3336
	Outdoor	2.87 ± 5.09	4.72 ± 7.46	32.3 (− 67.4, 72.6)	0.3986
Others	Indoor	0.034 ± 0.269	0.038 ± 0.302	NA*	NA*
	Outdoor	0.030 ± 0.196	0.037 ± 0.309	NA*	NA*

*Model-based analysis was not performed (a large amount 0' s); no statistically significant % change is expected

## Discussion

Temporal and routine indoor and outdoor HLC sampling over ten months of baseline and 25 months of intervention enabled the documentation of *Anopheles, Aedes, Armigeres* and *Culex* mosquitoes as well as the impacts of a spatial repellant product on entomological endpoints—primarily landing rates.

Baseline data demonstrate that the biting rates for *Anopheles* species and the other genera were balanced between intervention and placebo arms, with large amounts of variation seen both between houses within a cluster as well as between clusters. These variations may be due to variations in household levels of attractivity—based on the number of humans, household construction, number of animals, and proximity to larval sites [27]. Cluster 2 for example had the highest number of mosquitoes overall being surrounded by rice fields and other larval habitats. This large amount of variation resulted in a lack of statistical significance for most evaluations. Overall, the reduction in landing rates associated with the SR intervention compared with placebo houses was not statistically significant (16.4% indoors and 11.3% outdoors), and may be due to multiple factors including the presence of 13 *Anopheles* species with varying bionomic characteristics and varying densities between clusters.

The location of biting (indoor versus outdoor) was impacted by the SR intervention – demonstrated by the decrease in the biting rate indoors in the intervention arm relative to that in the placebo arm—0.79 bites indoors in the SR arm for every 1 bite indoors in the placebo arm. This was also seen in outdoor biting rates with there being 0.9 bites outdoors in the SR arm for every 1 bite in the placebo arm. The reduction in intervention and outdoor indoor biting rates demonstrates an overall SR-related reduction in landing, though, as would be expected, the reduction was higher indoors pointing to the efficacy of the indoor SR intervention. This was also supported when comparing indoor versus outdoor biting rates in the intervention clusters during the intervention. Here the placebo clusters had almost equal biting rates in both spaces but there were 0.89 bites indoors for every 1 bite outdoors in intervention clusters. The reduction in the outdoor biting rate extends the SR protective bubble outside the indoor protected spaces into outdoor spaces where there are limited interventions present, and where people tend to congregate in the evenings where transmission may occur. As reported in the primary manuscript, overall HLC outcomes were not statistically significant, but nevertheless demonstrated an epidemiological impact against malaria [19].

The HBR of three known *Anopheles* vectors—*An. flavirostris*, *An. leucosphyrus s.l.* and *An. maculatus,* impacted the malaria incidence in a statistically significant manner. All species with non-sparse data (with the exception of *An. sundaicus*) demonstrated an SR-based increase in PE, with five species demonstrating statistical significance. Here the higher numbers of mosquitoes caught, combined with species specific bionomic traits and the species specific impact of the intervention may have allowed for the relative impact. Though the reasons for the substantial decline in the *An. sundaicus* population over the course of this study remain unknown, this may have contributed to the lack of protective efficacy seen with this endophagic and anthropophagic species, and also explain the reduced overall reduction in SR-related biting rate seen here relative to earlier studies [18]. The relationship between almost all species and protective efficacy (with or without statistical significance) points to the impact of the SR intervention on a multitude of species with varying bionomic traits.

Overall, the SR intervention proportionally increased nulliparity in the sampled *Anopheles* populations compared to placebo both indoors and outdoors. The reduction in parous mosquitoes is an important outcome of the spatial repellant intervention pointing to effects other than repellency. Here, increased mortality in the local vector population exposed to the transfluthrin SR intervention will contribute to the overall protective efficacy by not just reducing landing, but also reducing the proportion of infections mosquitoes that do land. As expected, when parity was related to the hazard of malaria infection, the hazard of malaria infection increases when the parity increases, and decreases when nulliparity increases.

Though there were more sporozoite positive samples in the intervention arm, a rate of less than 0.5% for both arms pointed to the lack of a statistically significant difference between the SR arm when compared to the placebo—especially when the higher sporozoite rate seen here in the intervention arm is related to a lower incidence of disease. These low rates and lack of differences between arms question the value of these entomological indicators (including the entomological inoculation rate (EIR) in lower transmission settings [28].

Non-*Anopheles* mosquitoes were analysed by genus since only a subset were examined molecularly. Overall, just as with *Anopheles*, there was a demonstrated impact on landing rates both indoors and outdoors with large confidence intervals preventing significance. The impacts on non-*Anopheles* biting rates were higher than that seen in *Anopheles*, with the highest reduction see in endophilic *Aedes* (44% indoors and 35.6% outdoors). Similarly higher reductions were seen with *Armigeres* and *Culex.* The reductions in landing with these non-*Anopheles* is significant considering the diseases they transmit including dengue, Chikungunya, and Zika virus transmitted by *Aedes* species; filariasis transmitted by multiple genera of mosquito—*Mansonia*, *Anopheles*, *Culex*, *Aedes*, and *Armigere*s; and Japanese encephalitis (JE) transmitted by *Culex* species [1]. Reductions in these non-malaria mosquitoes with a malaria-centric intervention are also significant since, in addition to the potential impact on disease transmission, the perception of efficacy of an intervention by users is based on perceived biting from all mosquitoes and not just *Anopheles* vectors. Compliance and usage are a significant driver of intervention efficacy of interventions [29, 30] and the reduction in landing seen with nuisance mosquitoes (non-malaria vectors) points to a greater perception of efficacy, indicating higher use and an increased impact on protective efficacy.

This study documented a limited reduction (*Anopheles* HBR indoor: 16.4%; outdoor: 11.3%) in landing mosquitoes exposed to the active treatment compared to the placebo-control. The large variation seen between households and clusters, and the resulting wide confidence intervals resulted in low statistical significance. However, a statistically significant decrease on malaria infection related to the intervention was detected (60% protective efficacy) in these clusters [19]. This discrepancy of SR impact on malaria incidence and entomological correlates was also observed in another study, where a 32% reduction of *Anopheles* landing rates yielded a 52% reduction on malaria incidence [18]. The seemingly higher reduction in infections relative to the reduction in landing rates may indicate the accumulation of other SR impacts not limited to the reduction in landing. In addition to an impact on landing (repellency), exposure to the SR active may result in several other phenomena. These include feeding inhibition where a mosquito may land but not feed, as well as knock down with consequent delay in recovery and feeding and possibly death. Here, these SR-related impacts all directly impact disease transmission (reduce biting) and also increase the daily death rate thereby reducing the proportion of older and infectious females—documented by the decrease in parity in this study. These cumulative impacts would have the much higher impact documented relative to only describing landing rates. *Anopheles barbirostris* and the Leucosphyrus group are known filariasis vectors in rural area in many places of Indonesia—including Sumba. As with malaria, these cumulative impacts should also be seen with other diseases, as with non-malaria vectors as well.

## Conclusion

Overall, the SR intervention was documented to have an impact on landing rates in all anthropophagic mosquitoes, with the impact being measured on malaria. These results are encouraging and support the spatial repellant paradigm towards both malaria and other vector borne diseases. Documentation of vectors biting indoors and outdoors clearly indicates that currently implemented vector control tools that include provision of LLINs and IRS are not enough to protect local inhabitants from malaria (and other diseases transmitted in the peri-domestic area). Additional complementary vector control tools—such as LSM and SRs, targeting spaces and times where LLINs and IRS are not as efficacious would be necessary towards disease elimination.

## Supplementary Information

**Additional file 1.** Frequency (percentage) of *Anopheles* species.

**Additional file 2.** The relationship between HBR and malaria infection by species for overall infections.

**Additional file 3.** The mean (± SD) baseline HBR (bpn) of non-Anopheles mosquitoes.

## Data Availability

All relevant data are within the manuscript.

## Abbreviations

HBR: Human biting rates
s.l.: Sensu lato
s.s: Sensu stricto
SR: Spatial repellency
JE: Japanese encephalitis
API: Annual parasite incidence
LLINs: Long-lasting insecticidal nets
IRS: Indoor residual spraying
WHO: World Health Organization
GLP: Good laboratory practice
GCP: Good clinical practice
EC: Ethics Committee
HLC: Human landing catches
ITS2: Internal Transcribed Spacer 2
COI: Cytochrome C Oxidase subunit 1
PCR: Polymerase chain reaction
NB: Negative binomial
BH: Benjamini–Hochberg
MCP: Multiplicity adjustment procedure
SD: Standard deviation
BPN: Bites per person per night
EIR: Entomological inoculation rate
