# Supplementary material for: Impact of a spatial repellent product on Anopheles and non-Anopheles mosquitoes in Sumba, Indonesia
Source: Malar J. 2022 Jun 3;21:166. doi: 10.1186/s12936-022-04185-8 (PMC9166507; doi:10.1186/s12936-022-04185-8)
Supplement: Supplementary file 1 — Additional file 1. Frequency (percentage) of Anopheles species. [file 12936_2022_4185_MOESM1_ESM.docx]

| Anopheles Species | Baseline | | | | Intervention | | | |
| --- | --- | --- | --- | --- | --- | --- | --- | --- |
|  | Indoor | | Outdoor | | Indoor | | Outdoor | |
|  | SR | Placebo | SR | Placebo | SR | Placebo | SR | Placebo |
| **Total** | **2243 (100%)** | **3327 (100%)** | **2896 (100%)** | **3462 (100%)** | **3883 (100%)** | **4897 (100%)** | **4372 (100%)** | **4834 (100%)** |
| **aconitus** | **467 (20.82%)** | **80 (2.40%)** | **768 (26.52%)** | **78 (2.25%)** | **2015 (51.89%)** | **173 (3.53%)** | **2249 (51.44%)** | **162 (3.35%)** |
| **annularis** | **55 (2.45%)** | **633 (19.03%)** | **70 (2.42%)** | **567 (16.38%)** | **82 (2.11%)** | **544 (11.11%)** | **99 (2.26%)** | **493 (10.20%)** |
| balabacensis | 0 (0%) | 0 (0%) | 0 (0%) | 0 (0%) | 2 (0.05%) | 1 (0.02%) | 0 (0%) | 0 (0%) |
| **barbirostris** | **41 (1.83%)** | **148 (4.45%)** | **49 (1.69%)** | **132 (3.81%)** | **62 (1.60%)** | **360 (7.35%)** | **56 (1.28%)** | **322 (6.66%)** |
| barumbrosus | 0 (0%) | 0 (0%) | 0 (0%) | 0 (0%) | 1 (0.03%) | *0* (0%) | 0 (0%) | 3 (0.06%) |
| farauti | 0 (0%) | 0 (0%) | 0 (0%) | 0 (0%) | 0 (0%) | 1 (0.02%) | 1 (0.02%) | 0 (0%) |
| **flavirostris** | **355 (15.83%)** | **417 (12.53%)** | **523 (18.06%)** | **504 (14.56%)** | **757 (19.50%)** | **430 (8.78%)** | **1001 (22.90%)** | **482 (9.97%)** |
| **indefinitus** | 0 (0%) | 1 (0.03%) | 0 (0%) | 1 (0.03%) | 2 (0.05%) | 12 (0.25%) | 4 (0.09%) | 20 (0.41%) |
| karwari | 0 (0%) | 0 (0%) | 0 (0%) | 0 (0%) | 0 (0%) | 0 (0%) | 1 (0.02%) | 0 (0%) |
| **kochi** | **47 (2.10%)** | **501 (15.06%)** | **36 (1.24%)** | **392 (11.32%)** | **90 (2.32%)** | **803 (16.40%)** | **88 (4.71%)** | **836 (17.29%)** |
| leucosphyrus | 0 (0%) | 1 (0.03%) | 0 (0%) | 0 (0%) | 2 (0.05%) | 16 (1.61%) | 1 (0.02%) | 26 (0.54%) |
| **maculatus** | **61 (2.72%)** | **88 (2.65%)** | **95 (3.28%)** | **88 (2.54%)** | **209 (5.38%)** | **79 (1.61%)** | **206 (4.71%)** | **94 (1.94%)** |
| montanus | 0 (0%) | 0 (0%) | 0 (0%) | 0 (0%) | 1 (0.03%) | 0 (0%) | 1 (0.02%) | 0 (0%) |
| parangensis | 0 (0%) | 0 (0%) | 0 (0%) | 0 (0%) | 0 (0%) | 1 (0.02%) | 0 (0%) | 0 (0%) |
| punctulatus | 0 (0%) | 0 (0%) | 0 (0%) | 0 (0%) | 0 (0%) | 0 (0%) | 1 (0.02%) | 0 (0%) |
| sinensis | 0 (0%) | 0 (0%) | 0 (0%) | 0 (0%) | 0 (0%) | 4 (0.08%) | 0 (0%) | 2 (0.04%) |
| **subpictus s.l** | **15 (0.67%)** | **7 (0.21%)** | **13 (0.45%)** | **15 (0.43%)** | **3 (0.08%)** | **105 (2.14%)** | **10 (0.23%)** | **85 (1.76%)** |
| **sundaicus** | **1001 (44.63%)** | **401 (12.05%)** | **1154 (39.85%)** | **562 (16.23%)** | **136 (3.50%)** | **61 (1.25%)** | **128 (2.93%)** | **62 (1.28%)** |
| **tessellatus** | **94 (4.19%)** | **317 (9.53%)** | **89 (3.07%)** | **295 (8.52%)** | **205 (5.28%)** | **1246 (25.44%)** | **192 (4.39%)** | **1215 (25.13%)** |
| umbrosus | 0 (0%) | 0 (0%) | 1 (0.03%) | 0 (0%) | 0 (0%) | 0 (0%) | 0 (0%) | 0 (0%) |
| **vagus** | **107 (4.77%)** | **7,33 (22.03%)** | **98 (3.38%)** | **828 (23.92%)** | **315 (8.11%)** | **1061 (21.67%)** | **334 (7.64%)** | **1031 (21.33%)** |
| Unknown | 0 (0%) | 0 (0%) | 0 (0%) | 0 (0%) | 1 (0.03%) | 0 (0%) | 0 (0%) | 1 (0.02%) |
