# Supplementary material for: Impact of a spatial repellent product on Anopheles and non-Anopheles mosquitoes in Sumba, Indonesia
Source: Malar J. 2022 Jun 3;21:166. doi: 10.1186/s12936-022-04185-8 (PMC9166507; doi:10.1186/s12936-022-04185-8)
Supplement: Supplementary file 2 — Additional file 2. The relationship between HBR and malaria infection by species for overall infections. [file 12936_2022_4185_MOESM2_ESM.docx]

| Anopheles Spesies | Collection Location | Hazard Ratio | 95% CI | Raw 2-sided p-value | BH* adjusted p-value |
| --- | --- | --- | --- | --- | --- |
| aconitus | Indoor | 1.01 | (0.91, 1.11) | 0.902 | 0.966 |
|  | Outdoor | 0.96 | (0.87, 1.06) | 0.441 | 0.696 |
|  | Indoor + Outdoor | 0.96 | (0.88, 1.04) | 0.339 | 0.598 |
| annularis | Indoor | 0.92 | (0.81, 1.06) | 0.246 | 0.568 |
|  | Outdoor | 0.89 | (0.78, 1.02) | 0.091 | 0.341 |
|  | Indoor + Outdoor | 0.92 | (0.82, 1.03) | 0.139 | 0.348 |
| barbirostris | Indoor | 0.98 | (0.83, 1.16) | 0.851 | 0.946 |
|  | Outdoor | 0.92 | (0.76, 1.13) | 0.434 | 0.723 |
|  | Indoor + Outdoor | 0.98 | (0.84, 1.15) | 0.802 | 1.000 |
| flavirostris | Indoor | 1.15 | (1.06, 1.27) | 0.001 | **0.010** |
|  | Outdoor | 1.27 | (1.16, 1.38) | <0.0001 | **<0.003** |
|  | Indoor + Outdoor | 1.24 | (1.14, 1.36) | <0.0001 | **<0.003** |
| kochi | Indoor | 1.03 | (0.86, 1.24) | 0.727 | 0.948 |
|  | Outdoor | 1.00 | (0.81, 1.23) | 0.980 | 0.980 |
|  | Indoor + Outdoor | 1.05 | (0.89, 1.24) | 0.542 | 0.813 |
| leucosphyrus | Indoor | 0.69 | (0.44, 1.08) | 0.106 | 0.318 |
|  | Outdoor | 0.65 | (0.49, 0.85) | 0.002 | **0.015** |
|  | Indoor + Outdoor | 0.70 | (0.56, 0.89) | 0.003 | **0.018** |
| maculatus | Indoor | 0.95 | (0.90, 1.01) | 0.098 | 0.327 |
|  | Outdoor | 0.90 | (0.79, 1.03) | 0.138 | 0.376 |
|  | Indoor + Outdoor | 0.87 | (0.78, 0.98) | 0.016 | **0.080** |
| sundaicus | Indoor | 1.05 | (0.89, 1.23) | 0.592 | 0.846 |
|  | Outdoor | 1.02 | (0.86, 1.21) | 0.831 | 1.000 |
|  | Indoor + Outdoor | 1.04 | (0.97, 1.11) | 0.311 | 0.622 |
| tessellatus | Indoor | 1.00 | (0.88, 1.14) | 0.952 | 0.985 |
|  | Outdoor | 0.92 | (0.77, 1.08) | 0.306 | 0.656 |
|  | Indoor + Outdoor | 1.02 | (0.90, 1.16) | 0.719 | 0.980 |
| vagus | Indoor | 0.99 | (0.88, 1.11) | 0.847 | 0.977 |
|  | Outdoor | 0.90 | (0.80, 1.01) | 0.084 | 0.360 |
|  | Indoor + Outdoor | 0.95 | (0.86, 1.05) | 0.333 | 0.624 |
|  |  |  |  |  |  |
| *Benjamini-Hochberg multiplicity adjustment procedure | | | | |  |
| lnterpretation of hazard ratio (HR). with a e¹- 1 = 1.72 folds increase in HBR, the malaria incidence rate changes by (1-HR) x 100% | | | | | |
