# Supplementary material for: Impact of a spatial repellent product on Anopheles and non-Anopheles mosquitoes in Sumba, Indonesia
Source: Malar J. 2022 Jun 3;21:166. doi: 10.1186/s12936-022-04185-8 (PMC9166507; doi:10.1186/s12936-022-04185-8)
Supplement: Supplementary file 3 — Additional file 3. The mean (± SD) baseline HBR (bpn) of non-Anopheles mosquitoes. [file 12936_2022_4185_MOESM3_ESM.docx]

|  | Indoor | | Indoor | |
| --- | --- | --- | --- | --- |
|  | SR | Placebo | SR | Placebo |
|  | 7.30± 5.01 | 8.33 ± 5.95 | 7.32 ± 5.21 | 8.05 ± 5.73 |
| Aedes sp. | 2.48± 1.85 | 2.24± 2.47 | 2.34± 1.98 | 2.21± 2.34 |
| Armigeres | 0.46± 0.45 | 0.65± 0.39 | 0.49± 0.45 | 0.52± 0.36 |
| Culex sp. | 4.32± 3.90 | 5.70± 4.35 | 4.48± 4.30 | 5.30 ± 3.94 |
| Others | 0.020± 0.044 | 0.012± 0.032 | 0.002± 0.010 | 0.018± 0.034 |
